# Swarming Extraordinary

**Published:** 1880-12

**Authors:** 


### SWARMING EXTRAORDINARY.

D. N. Kern relates in the *Ohio Farmer* the following experience with a swarm of Italian bees:

The first swarm came out May 5, and was put in a hive filled with comb. On the 19th of May the second swarm came out, and was hived with a weak swarm. On the 20th the third came out, and was hived with the second and the weak swarm. On the 21st the fourth came out. Mr. Kern caught the queen and killed it, and put the swarm back to the old colony. On the 23d the fifth swarm came out. He caught two queens and killed them, and put the swarm back again. On the 24th, at nine o'clock A. M., the sixth swarm came out. He caught two queens again and killed them, and put the swarm back again. The same day, at three o'clock P. M., the seventh swarm came out again. This time he hived them in an old straw hive, and set them on top of the old hive. In the evening of the 25th he shook them down in front of the old hive again, and that settled for the time the swarming fever of the old hive. But on the 26th of June, the first young swarm threw out a very large swarm, and on July 3d threw out a second swarm, and about five minutes later a swarm came out of the old hive again. He hived both swarms together and sold them for $200 cash. All these swarms made 235 pounds of comb honey.

